# Prophage-Related Gene *VpaChn25_0724* Contributes to Cell Membrane Integrity and Growth of *Vibrio parahaemolyticus* CHN25

**DOI:** 10.3389/fcimb.2020.595709

**Published:** 2020-12-09

**Authors:** Lianzhi Yang, Yaping Wang, Pan Yu, Shunlin Ren, Zhuoying Zhu, Yinzhe Jin, Jizhou Yan, Xu Peng, Lanming Chen

**Affiliations:** ^1^ Key Laboratory of Quality and Safety Risk Assessment for Aquatic Products on Storage and Preservation (Shanghai), China Ministry of Agriculture, Shanghai, China; ^2^ College of Food Science and Technology, Shanghai Ocean University, Shanghai, China; ^3^ Department of Internal Medicine, Virginia Commonwealth University/McGuire VA Medical Centre, Richmond, VA, United States; ^4^ College of Fishers and Life Science, Shanghai Ocean University, Shanghai, China; ^5^ Archaea Centre, Department of Biology, University of Copenhagen, Copenhagen, Denmark

**Keywords:** *Vibrio parahaemolyticus*, prophage, virulence, gene knockout, secretome, transcriptome

## Abstract

*Vibrio parahaemolyticus* is a leading seafood-borne pathogen that can cause acute gastroenteritis and even death in humans. In aquatic ecosystems, phages constantly transform bacterial communities by horizontal gene transfer. Nevertheless, biological functions of prophage-related genes in *V. parahaemolyticus* remain to be fully unveiled. Herein, for the first time, we studied one such gene *VpaChn25_0724* encoding an unknown hypothetical protein in *V. parahaemolyticus* CHN25. This gene deletion mutant Δ*VpaChn25_0724* was constructed by homologous recombination, and its complementary mutant Δ*VpaChn25_0724*-com was also obtained. The Δ*VpaChn25_0724* mutant exhibited a sever defect in growth and swimming motility particularly at lower temperatures. Biofilm formation and cytotoxicity capacity of *V. parahaemolyticus* CHN25 was significantly lowered in the absence of *VpaChn25_0724*. Comparative secretomic analysis revealed an increase in extracellular proteins of Δ*VpaChn25_0724*, which likely resulted from its damaged cell membrane. Comparison of transcriptome data showed twelve significantly altered metabolic pathways in Δ*VpaChn25_0724*, suggesting inactive transport and utilization of carbon sources, repressed energy production and membrane biogenesis in *ΔVpaChn25_0724*. Comparative transcriptomic analysis also revealed several remarkably down-regulated key regulators in bacterial gene regulatory networks linked to the observed phenotypic variations. Overall, the results here facilitate better understanding of biological significance of prophage-related genes remaining in *V. parahaemolyticus*.

## Introduction

*Vibrio parahaemolyticus* is a gram-negative bacterium and thrives in marine, riverine, and aquaculture environments worldwide (Ghenem et al., 2017). Consumption of raw, undercooked or mishandled seafood contaminated by pathogenic *V. parahaemolyticus* can cause acute gastroenteritis in humans and even death (Kim et al., 2017). The bacterium was originally identified in 1950 in Osaka, Japan, where an outbreak of acute gastroenteritis, caused by contaminated semidried juvenile sardines, sickened 272 and killed 20 people (Fujino et al., 1953). Since then, the infectious disease caused by *V. parahaemolyticus* has been reported in many Asian countries, and subsequently in Africa, America, and Europe, arguing a pandemic of *V. parahaemolyticus* worldwide (Ghenem et al., 2017). In China, *V. parahaemolyticus* is a leading cause of foodborne bacterial disease, especially among adults in coastal regions in recent years (Jiang et al., 2019). Most pathogenic *V. parahaemolyticus* strains of clinical origin have two major virulence factors, a thermostable direct hemolysin (TDH) and a TDH-related hemolysin (TRH). Both toxins have hemolytic activity, enterotoxin activity, cardiotoxicity and cytotoxicity to the host (Li et al., 2019). Nevertheless, some *V. parahaemolyticus* isolates of environmental origins lacking the *tdh* and/or *trh* genes also show cytotoxicity to human intestinal cells, suggesting additional virulence-associated factors exist in the bacterium (Raghunath, 2014). Thus, identification of risk factors in *V. parahaemolyticus* is imperative for assuming food safety.

In aquatic ecosystems, phages are the most abundant biological entity involved in numerous biological cycles and constantly transform bacterial communities by horizontal gene transfer (HGT) (Penades et al., 2015; Castillo et al., 2018). Phage genomic DNA can integrate into and replicate as part of bacterial chromosomes, which is a typical feature of mild phages for a lysogen cycle (Feiner et al., 2015; Howard-Varona et al., 2017). HGT can result in pandemic or pathogenic clones with expanded ecological persistence and dispersibility, e.g., the filamentous phage VfO3:K6 in *V. parahaemolyticus* O3:K6 (Loyola et al., 2015). It constitutes important driving forces in host evolution, and bestows a wide range of phenotypes upon hosts with transmitted gene cassettes, such as phage-coded virulence, cell adhesion, antibiotic resistance, and metabolizing enzyme determinants (Harrison and Brockhurst, 2017; Castillo et al., 2018). Previous studies have revealed phages or prophage gene clusters present in *V. parahaemolyticus* (e.g., Jensen et al., 2013; Gomez-Gil et al., 2014; Kalburge et al., 2014). For instance, the phage Vp58.5 enhances ultraviolet sensitivity of *V. parahaemolyticus* O3:K6 (Zabala et al., 2009), and phage Vp882 transmits DNA adenine methylase and quorum sensing (QS) transcription factors to *V. parahaemolyticus* O3:K6 (Lan et al., 2009). A recently reported lytic *Vibrio* phage VP06 can infect a broad range of hosts, including *Vibrio alginolyticus, Vibrio azureus, Vibrio harveyi*, and *V. parahaemolyticus*. This *Vibrio* phage is resistant to environmental stresses, displaying potential as a candidate biocontrol agent (Wong et al., 2019).

In our prior studies, *V. parahaemolyticus* CHN25 strain (serotype: O5:KUT) was isolated, identified, and characterized (Song et al., 2013; Sun et al., 2014; He et al., 2015; Zhu et al., 2017). The complete genome sequence of *V. parahaemolyticus* CHN25 contains 5,443,401 bp with 45.2% G+C content (Zhu et al., 2017). Comparative genomic analysis revealed five prophage gene clusters in *V. parahaemolyticus* CHN25. The largest one has sequence similarity with a 33,277-bp *Vibrio* phage Martha 12B12 (GenBank accession no. HQ316581). Nevertheless, this prophage sequence exists in a truncated version in the bacterial genome, where approximately 37.5% of the remaining genes encode predicted proteins of unknown function. Identification of these hypothetical proteins is yet to be determined. We, therefore, asked whether *V. parahaemolyticus* CHN25 would be affected by the absence of these prophage-related genes. In this study, we aimed to address possible function of one such gene *VpaChn25_0724* in *V. parahaemolyticus* CHN25.

## Materials and Methods

### Bacterial Strains, Plasmids, and Culture Conditions

The *V. parahaemolyticu* CHN25 strain was used in this study. *Escherichia coli* DH5α λpir [BEINUO Biotech (Shanghai) Co. Ltd., China] was used as a host strain for DNA cloning. The pDS132 plasmid and *E. coli* β2155 λpir were used as a suicide vector and a donor strain in conjugation experiments, respectively (Zhu et al., 2017). The pMMB207 plasmid (Biovector Science Lab, Inc., China) was used as an expression vector to construct the reverse mutant. The *E. coli* strains were routinely incubated in Luria-Bertani (LB) medium (1% NaCl, pH 7.2) at 37°C, and the *V. parahaemolyticus* strains were grown in LB (3% NaCl, pH 8.5) or Tryptic Soy Broth (TSB) (3% NaCl, pH8.5) media. The growth medium was supplemented as needed with chloramphenicol to a final concentration of 30 μg/ml for *E. coli* and 5 μg/ml for *V. parahaemolyticu*s (Zhu et al., 2017).

### Construction of Deletion Mutant and Reverse Mutant of the VpaChn25_0724 Gene

Genomic DNA was prepared using TaKaRa MiniBEST Bacterial Genomic DNA Extraction Kit (Japan TaKaRa BIO, Dalian Company, China). Plasmid DNA was isolated using TIANpure Midi Plasmid Kit (Tiangen Biotech Beijing Co. Ltd., China). Oligonucleotide primers (**Table 1**) were designed using Primier 5.0 software (https://www.premierbiosoft.com), and synthesized by the Sangon Biotech (Shanghai) Co. Ltd., China. A markerless in-frame gene deletion mutant of the *VpaChn25_0724* gene was constructed using the homologous recombination method (Zhu et al., 2017). Briefly, based on the *VpaChn25_0724* gene sequence (294 bp) in *V. parahaemolyticus* CHN25 genome, the primer pairs (*VpaChn25_0724*-up-F/R and *VpaChn25_0724*-down-F/R) (**Table 1**) were designed to target the upstream (464 bp) and downstream (444 bp) sequences of the *VpaChn25_0724* gene, respectively. The amplified products by polymerase chain reaction (PCR) were individually digested with corresponding restriction endonucleases (TaKaRa, Japan), purified, and ligated into *XbaI* and *SacI* cloning sites on the pDS132. The ligated DNA was transformed into *E. coli* DH5α λpir competent cells and positive transformants were screened (Zhu et al., 2017). The recombinant plasmid pDS132+*VpaChn25_0724* was subsequently prepared and transformed into diaminopimelic acid (DAP) auxotroph *E. coli* β2155 competent cells grown in LB medium supplemented with 0.3 mM DAP (Sigma-Aldrich, USA). Plate mating assay was performed (Zhu et al., 2017). Exconjugants with successful double crossover deletions of the *VpaChn25_0724* gene were screened by colony PCR assay using the *VpaChn25_0724*-up-ex-F and *VpaChn25_0724*-down-ex-R primer pair (**Table 1**). The obtained Δ*VpaChn25_0724* mutant was confirmed by DNA sequencing, quantitative reverse transcription-PCR (RT-qPCR), and transcriptome analysis (see below). DNA sequences were determined by the Sangon (China).

**Table 1:** Oligonucleotide primers designed and used in this study.

Primer	Sequence (5′->3′)	Product size (bp)
*VpaChn25_0724*-up-F	GCTCTAGAATCGACCTATTCAGGC	464
*VpaChn25_0724*-up-R	TGGCGGCTCCATGAACCTCTATTTATC	
*VpaChn25_0724*-down-F	AGAGGTTCATGGAGCCGCCATGAAG	444
*VpaChn25_0724*-down-R	CGAGCTC TGGCGGCTTGCTCGATACGC	
*VpaChn25_0724*- up-ex-F	GAACTCGACCTGATATTG	1663
*VpaChn25_0724*-down-ex- R	CACATCCTCCTCAACCGC	
*Vpachn25_0724-*com-F	CGAGCTCATGTCCTTTAAAGATGTATTA	294
*Vpachn25_0724-* com-R	GCTCTAGATTACTTAGCGCGAGGGCGCTT	
*tlh-F*	AAAGCGGATTATGCAGAAGCACTG	596
*tlh-R*	ACTTTCTAGCATTTTCTCTGC	
*Vpachn25_0724* -F	ACCAGCGGTTAGTCATCTTG	154
*Vpachn25_0724* -R	ATTAGGCTTTGCTCTTCCAG	
*16s RNA-F *	GACACGGTCCAGACTCCTAC	179
*16s RNA-R*	GGTGCTTCTTCTGTCGCTAAC	
*VpaChn25_RS01720-F*	CTTAGCCACATCCCAACACC	196
*VpaChn25_RS01720-R*	TAGGACAAACAACCGCAATC	
*VpaChn25_RS03850-F*	ACCAGCGGTTAGTCATCTTG	154
*VpaChn25_RS03850-R*	ATTAGGCTTTGCTCTTCCAG	
*VpaChn25_RS04440-F*	ATGGGTCATCTTTATCTTTCG	183
*VpaChn25_RS04440-R*	CAGTCCGTTTAGCAGGTTCT	
*VpaChn25_RS06735-F*	GTAATAACCGACGCCTGCTC	165
*VpaChn25_RS06735-R*	ACGGGTGAATACGAAACGAA	
*VpaChn25_RS07910-F*	CTGCCGTGTTACCGATAAAG	184
*VpaChn25_RS07910-R*	CATCTCACCGCAATGAAAGC	
*VpaChn25_RS08070-F*	AGAACCAACTCTTAGGCTGGAC	114
*VpaChn25_RS08070-R*	TTAATGAACGCATTCGCTGT	
*VpaChn25_RS08820-F*	CAATCTTTAATTGCGTTGAG	144
*VpaChn25_RS08820-R*	AACCGATGTTCGTCACTATG	
*VpaChn25_RS11070-F*	GGTCTCGTTCATTGCACCTT	122
*VpaChn25_RS11070-R*	CTGCGGGTCTACAAATCTCG	
*VpaChn25_RS04175-F*	GACTAAACCGTATCGCTGAA	123
*VpaChn25_RS04175-R*	TGCCCATAGAAAGCATTACA	
*VpaChn25_RS13780-F*	GGTTTCGTTTAGGTCACG	277
*VpaChn25_RS13780-R*	ACGTCGAAATGTCGGCGG	
*VpaChn25_RS14070-F*	TGGTCGCGTAAGCAATGC	209
*VpaChn25_RS14070-R*	TTCGTCAGCTAGAGGAAG	

The underlined sequences represent the recognition sites of restriction endonucleases that were introduced via the forward and reverse primers, respectively.

The 294-bp *VpaChn25_0724* gene was amplified from the genomic DNA of *V. parahaemolyticus* CHN25 by PCR with the *VpaChn25_0724*-com-F/-R primers (**Table 1**). The PCR product was ligated into the expression vector pMMB207. The ligated DNA was transformed into *E. coli* DH5α and positive transformants were screened as described above. The recombinant plasmid pMMB207+*VpaChn25_0724* was then prepared and transformed into the Δ*VpaChn25_0724* mutant by electrotransformation as described previously (Zhu et al., 2017). The positive electrotransformants (Δ*VpaChn25_0724*-com mutant) were screened by colony PCR with primers *VpaChn25_0724*-com-F/R and *tlh*-F/R (**Table 1**), and confirmed using the aforementioned methods.

### Swimming Mobility and Biofilm Formation Assays

Swimming motility was examined according to the method described previously (Huang et al., 2017; Yang et al., 2017). Briefly, *V. parahaemolyticus* strains were individually incubated at 37°C in the TSB medium (pH 8.5, 3% NaCl) to the middle-logarithmic growth phase (mid-LGP) with OD_600nm_ values of about 0.8 to 1.0. Growth curves were measured using Bioscreen Automatic Growth Curve Analyzer (BioTek, USA). The differential growth phases were calculated on the basis of OD_600nm_ values between the wild type and mutant strains (Zhu et al., 2017). A 0.5 μl of each cell culture were inoculated into semi-solid TSB agar plates containing 0.25% agar, and incubated at 15°C, 25°C, and 37°C for 48, 24, and 12 h, respectively. The bacterial clones formed on the plates were measured and recorded.

Biofilm formation was quantified using the crystal violet staining method (Tan et al., 2018). Briefly, *V. parahaemolyticus* strains from overnight culture were individually diluted in the TSB medium to an absorption value at OD_600nm_ of about 0.4, and then 1 ml of the dilutions was individually inoculated into 24-well polystyrene microtiter plates (Sangon, China). After incubation at 37°C for 12 h, 24 h, 36 h, 48 h, and 60 h, the planktonic bacteria were removed, and biofilms were gently washed with 1 ml of 0.1M phosphate-buffered saline (PBS, pH 7.2 to 7.4, Sangon, China) for three times. The biofilms were then fixed using 0.1% (w/v) crystal violet (Sangon, China), washed, dissolved, and measured for absorbance values at OD_600nm_ using BioTek Synergy 2 (BioTek, USA) (Tan et al., 2018).

### Bacterial Cell Membrane Damage, Hydrophobicity, and Fluidity Assays

Cell membrane damage was analyzed using the method described by Collado et al. (Collado et al., 2017). The bacterial cell suspension was double dyed using propidium iodide (PI) (final concentration 10 mM) (Sangon, China) and 5(6)-carboxydiacetate fluorescein succinimidyl ester (CFDA) (final concentration 10 μM) (Beijing Solarbio Science & Technology Co. Ltd., China). Bacterial cell forward scatter, lateral astigmatism, and fluorescent channels FL1 (green) and FL2 (red) were determined using a flow cytometer BD FACSVerse™ (Becton, Dickinson and Company, USA). 1,000 cells were detected in each sample.

The cell membrane hydrophobicity and fluidity assays were performed as described by Pelletier et al. (Pelletier et al., 1997), and Voss and Montville (Voss and Montville, 2014), respectively.

### Secretome Analysis

Extracellular proteins of *V. parahaemolyticus* strains were extracted according to the method described previously (He et al., 2015). The 2-dimensional gel electrophoresis (2-DE) was performed according to the method by Zhu et al. (Zhu et al., 2020). Briefly, approximately 20 μg of extracellular proteins was diluted with the rehydration buffer [8 M urea, 4% (w/v) CHAPS, 65 mM dithiothreitol, 0.2% (vol/vol) Bio-Lyte 3/10 ampholyte and 0.0001% (wt/vol) bromophenol blue (Sangon, China)] to a final volume of 200 μl per sample. The mixture of each sample was applied to the pH gradient gel (IPG) strips (pH 4–7, 7 cm, Bio-Rad, USA) and passive rehydrated for 16 h at 17°C. After rehydration, IEF (isoelectric focusing) was run with a six-step program (Zhu et al., 2020). Following the electrophoresis in the first dimension, the strips were first equilibrated for 15 min in equilibration buffer I, and then washed for a further 15 min with equilibration buffer II (Zhu et al., 2020). The second-dimension separation was performed using SDS-polyacrylamide gel electrophoresis (SDS-PAGE). The strips were individually transferred onto 12.5% separation gel using a Mini-PROTEANW electrophoresis cell (Bio-Rad, USA) with a 2-step program (Zhu et al., 2020). The gels were stained, imaged, and analyzed as described previously (He et al., 2015). Additionally, amino acid sequences of protein spots were determined using liquid chromatography tandem mass spectrometry (LC-MS/MS) technique at Shanghai Houji Biology Co Ltd., China as described previously (Zhu et al., 2020).

### Human Intestinal Epithelial Cell Viability and Apoptosis Assay

Human intestinal epithelial cell viability infected by *V. parahaemolyticus* strains was determined as described previously (Tsai et al., 2018) with minor modifications. Human rectal cancer epithelial cell line Caco-2 (ATCC number : HTB-37™) was purchased from Stem Cell Bank, Chinese Academy of Sciences (Shanghai, China). Briefly, Caco-2 cells were seeded into 96-well cell culture plates at a concentration of 5 × 10^4^ cells/ml per well, and cultured in Dulbecco’s modified eagle medium (DMEM, Gibco, USA) at 37°C, 5% CO_2_ for 24 h using a CO_2_ Cell Incubator (Thermo, USA). The cell culture fluid was aspirated off, and cells were washed twice with 0.1 M PBS (pH 7.2–7.4, Sangon, China). Meanwhile, *V. parahaemolyticus* strains grown to the mid-LGP at 37°C were individually harvested, and cell pellet was washed twice with 0.1 M PBS, and resuspended with the phenol red-free DMEM medium to adjust OD_490nm_ values of about 0.2 ± 0.02. A 100 μl of the bacterial suspension was added into each well containing the Caco-2 cells, and 10 μl of [2-(2-methoxy-4-nitrophenyl)-3-(4-nitrophenyl)-5-(2,4-disulfonate)-2h-tetrazole monosodium salt (CCK-8, Sigma-Aldrich, USA)] was also added and incubated for 4 h at 5% CO_2_, 37°C. Caco-2 cell viability was calculated according to the following formula: cell viability (%) = [A (bacteria) − A (blank)]/[A (0 bacteria) − A (blank)] × 100, in which A (bacteria) represents an absorbance at OD_450nm_ of the cell culture wells with Caco-2 cells, CCK-8 solution and bacterial suspension; A (blank) represents a OD_450nm_ value without Caco-2 cells, DME medium and CCK-8 solution; A (0 bacteria) represents a OD_450nm_ value with Caco-2 cells and CCK-8 solution but without bacterial suspension.

A 2 ml of Caco-2 cells (1 × 10^5^ cells/ml) was inoculated into 6-well cell culture plates, and individually infected by *V. parahaemolyticus* strains for 4 h at 5% CO_2_, 37°C. Then a 200-μl trypsin (Gibco, USA) was added into each cell culture well to digest for 3 min. Then the supernatant was discarded, and cells were collected by centrifugation at 800 rpm for 4 min. Apoptosis of the Caco-2 cells was assayed using Annexin-V-FITC/PI Apoptosis Detection Kit (Solarbio, China), according to the manufacturer’s protocol. The treated samples were detected by BD FACSVerse™ flow cytometry (Becton, Dickinson and Company, USA), and data analysis was performed using FlowJo software (https://www.flowjo.com).

### Illumina RNA Sequencing

Total RNA was prepared using RNeasy Protect Bacteria Mini Kit (QIAGEN Biotech Co. Ltd., Germany) and QIAGEN RNeasy Mini Kit (QIAGEN) according to the manufacturer’s protocols. The DNA was removed from the samples using RNase-Free DNase Set (QIAGEN). Three independently prepared RNA samples were used in each Illumina RNA-sequencing experiment. The sequencing library construction and Illumina sequencing were conducted at Shanghai Majorbio Bio-pharm Technology Co. Ltd., China using Illumina HiSeq 2500 platform as described previously (Zhu et al., 2017). High quality reads that passed the Illumina quality filters were used for sequence analyses.

### Real-Time Reverse Transcription-PCR Assay

The RT-qPCR was performed as described previously (Wang et al., 2013). *V. parahaemolyticus* cultures grown to the mid-LGP were harvested for RNA extraction. The RNeasy Mini Kit (Qiagen, Germany) was used to extract total RNA. Reverse transcription reactions were performed using PrimeScript™ RT reagent Kit with gDNA Eraser (Perfect Real Time) (TaKaRa, Japan) kit. Relative quantitative PCR reactions were performed with TB Green^®^ Premix Ex Taq™ II (Tli RNaseH Plus) (TaKaRa, Japan) kit using 7500 Fast Real-Time PCR Instrument (Applied Biosystems, USA). The 16S rRNA gene was used as the internal reference gene, and 2^−ΔΔCt^ method was used to calculate the relative expression between the target and the internal reference genes. The representative eighteen differentially expressed genes (DEGs) in the transcriptome of Δ*VpaChn25_0724* mutant were confirmed by the RT-qPCR assay, and listed in **Table S1**.

### Detection of VpaChn25_0724 Gene by PCR

Total 138 V*. parahaemolyticus* strains isolated from commonly consumed aquatic products collected in Shanghai, China (Su et al. unpublished) were used in this study. The *VpaChn25_0724* gene was amplified with the primers *VpaChn 25_0724* –F/R (**Table 1**) by PCR (Song et al., 2013). A 6 μl of each PCR product was analyzed by agarose gel (1%) electrophoresis, imaged and recorded, and validated by DNA sequencing as described above.

### Transmission Electron Microscope Assay

The TEM observation of *V. parahaemolyticus* strains was conducted using ultrathin edge-cutting method (Morgelin, 2017; Xu et al., 2017) *via* a transmission electron (JEM2100, JEOL, Japan, ×80,000) at the Instrumental Analysis Center at Shanghai Jiao Tong University, Shanghai, China.

### Enzyme Activity Assays

The wild type and mutant strains grown to the mid-LGP were individually harvested by centrifugation at 8,000 g for 10 min at 4°C. The supernant was removed, and the bacterial cell pellets were used for the malate dehydrogenase and citrate synthase activity assays using the corresponding kits (Product Nos. BC1040, and BC1060, Beijing Solarbio Science & Technology Co. Ltd., China), according to the manufecturer’s instructions. Approximately 0.05 ± 0.002 g and 0.1 ± 0.02 g of each sample was used in the malate dehydrogenase and citrate synthase reactions, and their OD_340nm_ and OD_412nm_ values were recorded using BioTek Synergy 2 (BioTek, USA), respectively.

### Data Analysis

Quality filtration of raw RNA-seq data were performed using theSeqPrep (https://github.com/jstjohn/SeqPrep) and Sickle version 1.33 software (https://github.com/najoshi/sickle) as described previously (Zhu et al., 2017). The resulting clean reads were aligned to the *V. parahaemolyticus* CHN25 genome using the Bowtie2 version 2.0.5 software (http://bowtie-bio.sourceforge.net/bowtie2/index.shtml). Expression of each gene was calculated using RNA-Seq by Expectation-Maximization (RSEM, http://deweylab.github.io/RSEM/). Genes with the criteria, fold-changes ≥ 2.0 or ≤ 0.5, and p-values by BH (fdr correction with Benjamini/Hochberg) < 0.05 relative to the control, were defined as DEGs. These DEGs were used for gene set enrichment analysis (GSEA) against the Kyoto Encyclopedia of Genes and Genomes (KEGG) database (http://www.genome.jp/kegg/) and gene set annotation analysis (GSAA) against the Gene Ontology (GO) database (http://www.geneontology.org) as described previously (Zhu et al., 2017). Significantly changed GSEA were identified when the enrichment test p-value fell below 0.05.

Prophage gene clusters were searched and analyzed using Prophage Finder (http://phast.wishartlab.com/) and Basic Local Alignment Search Tool (BLAST) (http://www.ncbi.nlm.nih.gov/BLAST) software. All tests in this study were conducted in at least triplicate. The data were analyzed using SPSS statistical analysis software version 17.0 (SPSS Inc., USA).

## Results

### Gene Organization of the Largest Prophage Gene Cluster in Vibrio parahaemolyticus CHN25 Genome

**Figure 1:**
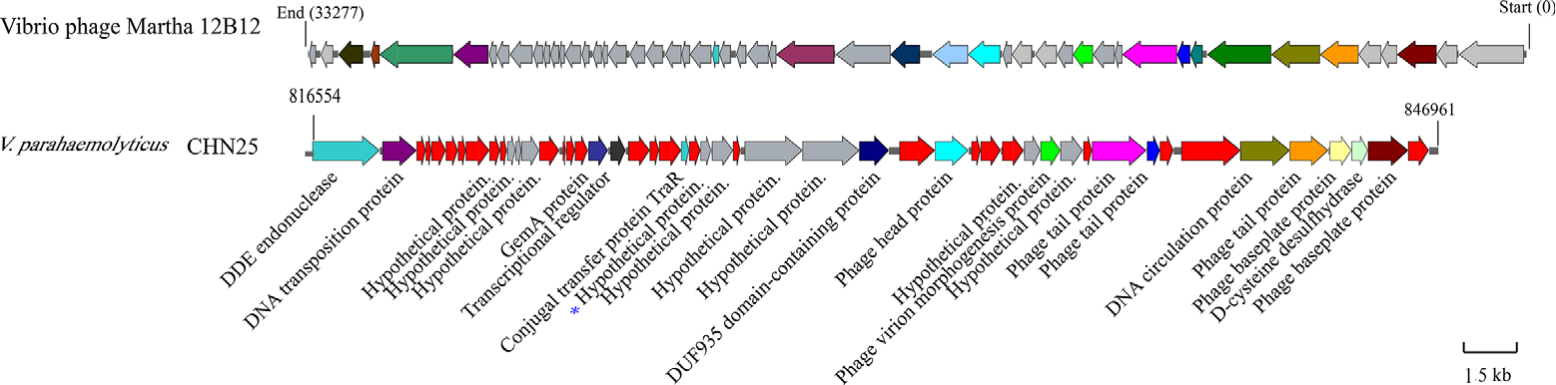
Gene organization of the *Vibrio* phage Martha 12B12-like sequence in chromosome 1 of *V. parahaemolyticus* CHN25 genome. Genes in gray color represent predicted hypothetical proteins, and those in red represent additional proteins absent from the *Vibrio* phage Martha 12B12. The *VpaChn25_0724* gene was marked with a star in blue.

Comparative genomic analysis revealed that the largest prophage gene cluster in *V. parahaemolyticus* CHN25 genome has sequence similarity to the *Vibrio* phage Martha 12B12 that contains 50 predicted genes. Approximately 24 genes thereof were present in chromosome 1 (3,416,467 bp) of *V. parahaemolyticus* CHN25 genome, where they located in the locus from 816,554 bp to 846,961 bp (**Figure 1**). Among the 24 genes, 7 coded for potential phage proteins (i.g., phage head, tail, and baseplate), 8 for predicted regulators, and 9 for hypothetical structural proteins of unknown function. Within the gene cluster, genes encoding DNA endonuclease, DNA transport protein, conjugal transfer protein TraR, and additional proteins were also identified, suggesting that the bacterium underwent extensive genetic recombination *via* HGT during its evolution (Zhu et al., 2017). Among the genes encoding unknown hypothetical proteins, the 294-bp *VpaChn25_0724* gene was further investigated and reported in this study.

### Deletion and Reverse Complementation of the VpaChn25_0724 Gene

To study biological function of the *VpaChn25_0724* gene, we constructed an unmarked in-frame gene deletion mutant Δ*VpaChn25_0724* using the homologous recombination method (**Figure S1**). The upstream and downstream sequences (approximately 0.5 kb) that flank the *VpaChn25_0724* gene were obtained by PCR (**Table 1**), and then cloned into a suicide vector pDS132 to yield a recombinant vector pDS132+*VpaChn25_0724*. The 908-bp inserted sequence was validated by DNA sequencing (data not shown). The recombinant vector was transformed into *E. coli* β2155, and the chloramphenicol-resistant transformant was obtained and conjugated with *V. parahaemolyticus* CHN25. Positive exconjugants were obtained using the two-step allelic exchange method. Deletion of the 294-bp *VpaChn25_0724* gene from *V. parahaemolyticus* CHN25 genome was validated by PCR and DNA sequencing assays (data not shown), as well as by RT-qPCR and transcriptomic analysis (see below).

To facilitate a complementation assay, a reverse mutant Δ*VpaChn25_0724*-com was also successfully constructed. The 294-bp *VpaChn25_0724* gene was amplified from genomic DNA of *V. parahaemolyticus* CHN25 by PCR, and then cloned into an expression vector pMMB207, which yielded a recombinant vector pMMB207-*VpaChn25_0724*. The inserted 294-bp sequence was confirmed by DNA sequencing (data not shown). This recombinant vector was then electrotransformed into the Δ*VpaChn25_0724* mutant, and generated the reverse mutant Δ*VpaChn25_0724*-com that was further confirmed using the aforementioned methods.

### Growth of the ΔVpaChn25_0724 Mutant at Different Temperatures

**Figure 2:**
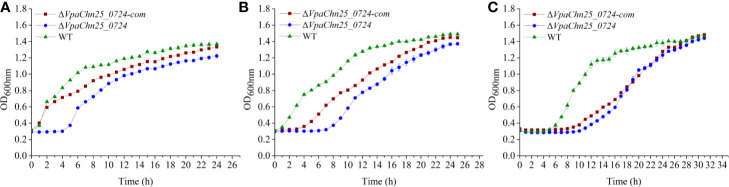
Survival of *V. parahaemolyticus* CHN25 (WT), Δ*VpaChn25_0724*, and Δ*VpaChn25_0724*-com strains at different temperatures. The strains were incubated in the TSB medium at 37°C **(A)**, 25°C **(B)**, 15°C **(C)**, respectively. WT, wild type.

To gain insights into possible impact of the *VpaChn25_0724* gene deletion on bacterial growth, we determined growth curves of *V. parahaemolyticus* CHN25, Δ*VpaChn25_0724*, and Δ*VpaChn25_0724*-com strains at 37°C, 25°C, and 15°C, which are within the temperature range experienced by *V. parahaemolyticus* during its life cycle (Desai and Kenney, 2019). As illustrated in **Figure 2**, the Δ*VpaChn25_0724* mutant exhibited a significantly extended lag phase, which was 4-fold, 6-fold, and 2.5-fold of that of the wild type strain at 37°C, 25°C, and 15°C, respectively. Complementation with a plasmid-borne *VpaChn25_0724* (Δ*VpaChn25_0724*-com) restored the growth almost completely at 37°C and partially at 25°C, but not at all at 15°C. These results highlighted the importance of *VpaChn25_0724* gene for the growth of *V. parahaemolyticus* CHN25, particularly at the lower temperatures.

### Swimming Motility and Biofilm Formation of the ΔVpaChn25_0724 Mutant

**Figure 3:**
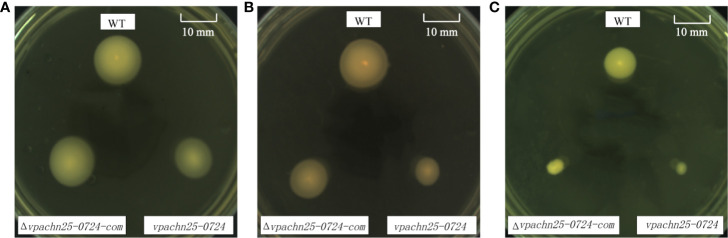
Swimming motility of *V. parahaemolyticus* CHN25 (WT), Δ*VpaChn25_0724*, and Δ*VpaChn25_0724*-com strains at different temperature. A: 37°C; B: 25°C; C: 15°C; WT: wild type. The strains were incubated on the semi-solid TSB agar plates containing 0.25% agar at 37°C **(A)**, 25°C **(B)**, 15°C **(C)**, respectively. WT, wild type.

As shown in **Figure 3**, on the semi-solid swimming plates, the lower temperatures notably shrunk swimming cycles of wild type, Δ*VpaChn25_0724*, and Δ*VpaChn25_0724*-com strains. However, swimming capacity of the wild type was about 2-fold and 3-fold higher than that of the Δ*VpaChn25_0724* mutant at 25°C and 15°C, respectively (p<0.05). No significant difference in swimming cycles was observed among the three strains at 37°C (p > 0.05). Additionally, the lower temperatures appeared to inhibit restoring the different phenotypes by the Δ*VpaChn25_0724*-com strain. Therefore, we focused on the optimal growth temperature 37°C in the following analysis in this study. These results indicated that the severe defect in growth elicited by the *VpaChn25_0724* gene deletion likely led to the variant swimming motility of *V. parahaemolyticus* CHN25 at the lower temperatures.

**Figure 4:**
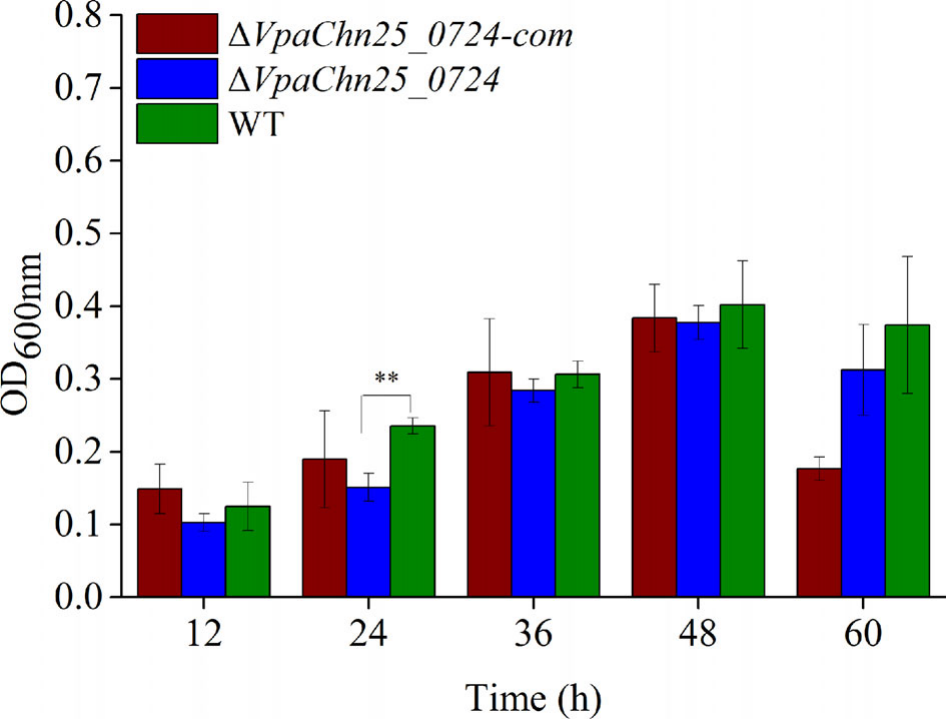
Biofilm formation of *V. parahaemolyticus* CHN25 (WT), Δ*VpaChn25_0724*, and Δ*VpaChn25_0724*-com strains. The strains were incubated in the TSB medium at 37°C under static conditions. WT: wild type. **p<0.01 compared with the WT.

In addition, we analyzed and quantified the bacterial bioﬁlms formed under static incubation conditions using the crystal violet staining assay. The dynamic process of biofilm formation was followed for the wild type, Δ*VpaChn25_0724*, and Δ*VpaChn25_0724*-com strains grown at 37°C for 60 h (**Figure 4**). We observed that biofilms were built at three different stages (development, maturation and diffusion) by all the three strains, consistent with previous studies. At the early formation stage (24 h), the Δ*VpaChn25_0724* mutant exhibited a 1.5-fold decrease in biomass compared with the wild type (p<0.01). No significant difference was observed at the other stages among the three strains (p>0.05), except a notably decrease in biomass of Δ*VpaChn25_0724*-com at the latter stage. These results showed a defect in early biofilm formation of *V. parahaemolyticus* CHN25 in the absence of the *VpaChn25_0724* gene.

### Cell Membrane Damage, Hydrophobicity, and Fluidity of the ΔVpaChn25_0724 Mutant

**Figure 5:**
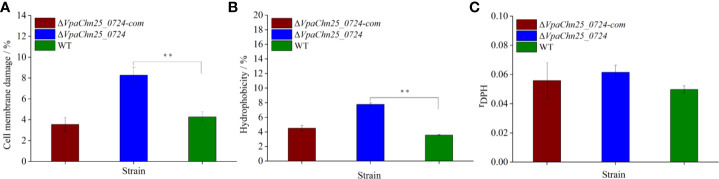
Cell membrane damage **(A)**, hydrophobicity **(B)** and fluidity **(C)** of *V. parahaemolyticus* CHN25 (WT), Δ*VpaChn25_0724*, and Δ*VpaChn25_0724*-com. The strains were incubated in the TSB medium at 37°C to the mid-LGP. WT: wild type. **p<0.01 compared with the WT.

On the basis of the above results, we, therefore, asked whether bacterial cell membrane structure would be affected by the *VpaChn25_0724* gene deletion. We evaluated cell membrane damage, hydrophobicity and fluidity of the three strains grown in the TSB medium to mid-LGP at 37°C. As shown in **Figure 5**, no significant difference in cell membrane fluidity was observed among the three strains (p>0.05). However, the proportion of Δ*VpaChn25_0724* cells with damaged membrane was about twofold of that of the wild type (p < 0.01). The Δ*VpaChn25_0724* mutant also showed a twofold cell surface hydrophobicity (p<0.01) in comparison to the wild type. In correlation to the almost full restoration of cellular growth by the plasmid-borne *VpaChn25_0724* at 37°C (**Figure 1**), the complementation also restored the membrane integrity and hydrophobicity phenotypes. These results indicated that the *VpaChn25_0724* gene is important for cell membrane integrity of *V. parahaemolyticus* CHN25.

### Differential Secretomes Mediated by the VpaChn25_0724 Gene Deletion

**Figure 6:**
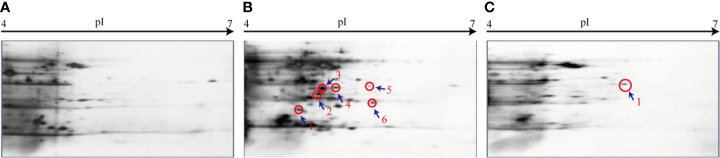
The 2-DE analysis of extracellular proteins of *V. parahaemolyticus* CHN25, Δ*VpaChn25_0724*, and Δ*VpaChn25_0724*-com strains. **(A)** Wild type; **(B)** Δ*VpaChn25_0724*; **(C)** Δ*VpaChn25_0724*-com.

Given the altered cell membrane trait mediated by the *VpaChn25_0724* gene deletion, we next conducted comparative secretomic analysis of the three strains. When incubated in the TSB medium at 37°C without shaking, the Δ*VpaChn25_0724* mutant also grew more slowly than wild type (data not shown). The supernatant of bacterial cultures at the mid-LGP were collected, and extracellular proteins were isolated, and analyzed by 2-DE assay. This analysis revealed different secretome profiles among the wild type, Δ*VpaChn25_0724* and Δ*VpaChn25_0724*-com strains, showing various numbers of visible protein spots (**Figure 6**). The patterns yielded from three independent 2-DE gels per biological sample were consistent (data not shown). These varied protein spots were excised from the 2-DE gels and digested with the trypsin. The resulting peptides were further identified by LC-MS/MS analysis.

The results yielded from the LC-MS/MS analysis were summarized in **Table 2**. Sequences of seven differentially expressed extracellular proteins among the three strains were obtained. Six proteins thereof were secreted by the Δ*VpaChn25_0724* mutant. For instance, the protein Spot 24-b-1 was identified as an 8-stranded β-barrel protein (OmpW). The protein Spots 24-b-2 and 24-b-3 were identified as FlaB/D and FlaA flagellins, respectiely, while the 24-b-6 and 24-C-1 were identified as an aldehyde-alcohol dehydrogenase (AdhE), and a 2-hydroxyacid dehydrogenase, respectively. These results indicated that the *VpaChn25_0724* gene deletion resulted in increased numbers of extracellular proteins, which was likely associated with damaged cell membrane structure of *V. parahaemolyticus* CHN25.

**Table 2:** Identification of the protein spots on the secretome profiles by LC-MS/MS analysis.

Protein spot	Uniprot No.	Protein	Gene	MW (Da)	pI	Score	Sequence coverage
24-b-1	Z2ENQ0	Outer membrane protein	*ompW*	23467.38	4.98	34.42	6.54%
24-b-2	A6BAT3	Polar flagellin B/D	*A79_3829*	39327.82	5.01	64.04	3%
24-b-3	C8CP39	Flagellin flaA	*flaA*	39776.44	4.9	63.57	5.32%
24-b-4	A0A2R9VMM8	Phage head morphogenesis protein	*C1S91_15620*	48343.9	4.79	23.63	1.62%
24-b-5	S5IY46	D-lactate dehydrogenase	*M634_18815*	36750.66	5.6	30.14	2.72%
24-b-6	A0A0M9C4Z3	Aldehyde-alcohol dehydrogenase	*ACX03_17620*	97061.86	5.68	34.29	1.11%
24-c-1	A0A0L8BEZ4	2-hydroxyacid dehydrogenase	*C9I78_22190*	36706.56	5.49	23.88	2.72%

### Effects of the VpaChn25_0724 Gene Deletion on V. parahaemolyticus CHN25-Host Intestinal Epithelial Cell Interaction

**Figure 7:**
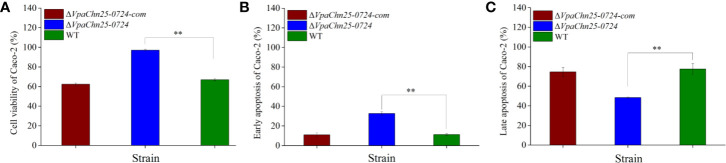
The viability and apoptosis of Caco-2 cells infected by *V. parahaemolyticus* CHN25 (WT), Δ*VpaChn25_0724*, and Δ*VpaChn25_0724-com* strains. The infection was performed at 37°C for 4 h. **(A)** Cell viability; **(B)** Early stage of apoptosis; **(C)** Late stage of apoptosis. **p<0.01 compared with the WT.

Consequently, we reasoned that the changed secretome may affect *V. parahaemolyticus* CHN25-host intestinal epithelial cell interaction, whereby the bacterium elicits gastroenteritis disease (O’Boyle and Boyd, 2013). The human rectal cancer epithelial cell line Caco-2 was used as an *in vitro* model for the cell interaction analysis in this study. As shown in **Figure 7**, after infected with the *ΔVpaChn25_0724* mutant at 37°C for 4 h, the viability of Caco-2 cells was significantly higher (97.05% ± 0.84) than those infected with the wild type (66.97% ± 1.04) and Δ*VpaChn25_0724*-com (62.38% ± 1.34) strains (p<0.01). Moreover, apoptosis of Caco-2 cells was examined using Annexin V-FITC and propidium iodide (PI) double stainings by flow cytometry assay. Unexpectedly, the results showed that at 4 h post infection, *ΔVpaChn25_0724* induced an early apoptosis in Caco-2 cells at a much higher rate (32.67% ± 2.12%) than the wild type (11.27% ± 0.94) and the Δ*VpaChn25_0724*-com (10.94% ± 2.12%) strains (p<0.01). Interestingly, an opposite pattern was observed where late apoptosis elicited by *ΔVpaChn25_0724* was significantly less (48.50% ± 0.30%) than by wild type (77.53% ± 5.59%) and Δ*VpaChn25_0724*-com (74.57% ± 4.56%) strains (p<0.01).

### Differential Transcriptomes Mediated by the VpaChn25_0724 Gene Deletion

To get insights into global-level gene expression change mediated by the *VpaChn25_0724* gene deletion, we next determined transcriptomes of *V. parahaemolyticus* CHN25, *ΔVpaChn25_0724* and Δ*VpaChn25_0724*-com strains using Illumina RNA sequencing technique. This analysis revealed that approximately 13.7% of* *the bacterial genes were differentially expressed in the *ΔVpaChn25_0724* mutant, when compared with the wild type and *ΔVpaChn25_0724-*com strains grown at 37°C to the mid-LGP. Of these genes, 190 showed higher transcriptional levels (fold change ≥ 2.0), while 569 genes were down-regulated (fold change ≤ 0.5). These DEGs in *ΔVpaChn25_0724* were grouped into one hundred and twenty-six gene functional catalogues in the KEGG database (data not shown). A complete list of the DEGs in the three strains is available in the NCBI SRA database (http://www.ncbi.nlm.nih.gov/sra/) under the accession number SRP258529. To validate the transcriptome data, we examined 18 representative genes (**Table S1**) in the *ΔVpaChn25_0724* mutant by RT-qPCR analysis. The resulting data were correlated with those yielded from the transcriptome analysis (**Table S1**).

### The Major Altered Metabolic Pathways in the ΔVpaChn25_0724 Mutant

Based on the GSEA of the transcriptome data against the KEGG database, approximately twelve significantly altered metabolic pathways were identified in the *VpaChn25_0724* mutant, including the galactose, glyoxylate and dicarboxylate, fructose and mannose, butanoate and thiamine metabolisms; citrate cycle (TCA); pentose and glucuronate interconversions; valine, leucine, and isoleucine degradation and glycerolipid metabolism; QS; ATP-binding cassette (ABC) transporters; and phosphotransferase system (PTS) (**Table 3**).

**Table 3:** Major altered metabolic pathways in the *ΔVpaChn25_0724* mutant.

Metabolic pathway	Gene ID	Fold change	Description
Galactose metabolism	*VpaChn25_RS11745*	0.441	Galactose-1-epimerase
	*VpaChn25_RS11750*	0.318	Galactokinase
	*VpaChn25_RS11755*	0.215	UDP-glucose-hexose-1-phosphate uridylyltransferase
	*VpaChn25_RS11760*	0.376	UDP-glucose 4-epimerase GalE
	*VpaChn25_RS11770*	0.403	beta-galactosidase subunit alpha
	*VpaChn25_RS21125*	0.185	beta-galactosidase
	*VpaChn25_RS06235*	0.452	alpha-galactosidase
	*VpaChn25_RS21110*	0.167	UDP-glucose 4-epimerase GalE
	*VpaChn25_RS11775*	0.406	beta-galactosidase subunit beta
	*VpaChn25_RS21100*	0.233	Galactokinase
	*VpaChn25_RS21105*	0.171	UDP-glucose–hexose-1-phosphate uridylyltransferase
Fructose and mannose metabolism	*VpaChn25_RS19530*	0.326	Fused PTS fructose transporter subunit IIA/HPr protein
	*VpaChn25_RS21730*	0.273	PTS mannitol transporter subunit IICB
	*VpaChn25_RS21735*	0.156	PTS mannitol transporter subunit IIA
	*VpaChn25_RS21725*	0.276	L-sorbose 1-phosphate reductase
	*VpaChn25_RS22280*	0.244	PTS fructose transporter subunit IIC
	*VpaChn25_RS22285*	0.129	Mannose-6-phosphate isomerase%2C class I
	*VpaChn25_RS01915*	0.191	Phosphoenolpyruvate–protein phosphotransferase
	*VpaChn25_RS20215*	0.385	Mannose-6-phosphate isomerase%2C class I
	*VpaChn25_RS19525*	0.307	1-phosphofructokinase
	*VpaChn25_RS17315*	0.254	PTS sugar transporter subunit IIA
	*VpaChn25_RS19520*	0.310	PTS fructose transporter subunit IIBC
Glyoxylate and dicarboxylate metabolism	*VpaChn25_RS02855*	3.967	Malate synthase A
	*VpaChn25_RS12740*	0.409	Dihydrolipoyl dehydrogenase
	*VpaChn25_RS07820*	0.393	Twin-arginine translocation signal domain-containing protein
	*VpaChn25_RS07825*	0.305	4Fe-4S dicluster domain-containing protein
	*VpaChn25_RS07830*	0.413	Formate dehydrogenase subunit gamma
	*VpaChn25_RS16830*	2.986	Malate synthase
	*VpaChn25_RS22250*	0.484	Catalase
	*VpaChn25_RS02860*	2.131	Isocitrate lyase
	*VpaChn25_RS18565*	2.087	Thiolase family protein
	*VpaChn25_RS01720*	0.420	Malate dehydrogenase
	*VpaChn25_RS16175*	0.465	Bifunctional 4-hydroxy-2-oxoglutarate Aldolase/2-dehydro-3-deoxy-phosphogluconate aldolase
	*VpaChn25_RS13855*	2.071	Alanine–glyoxylate aminotransferase family protein
Citrate cycle	*VpaChn25_RS12750*	0.421	Pyruvate dehydrogenase (acetyl-transferring)%2C homodimeric type
	*VpaChn25_RS09275*	0.486	Fumarate hydratase
	*VpaChn25_RS04480*	0.475	Succinate–CoA ligase subunit alpha
	*VpaChn25_RS00605*	0.365	Phosphoenolpyruvate carboxykinase (ATP)
	*VpaChn25_RS12745*	0.398	Pyruvate dehydrogenase complex dihydrolipoyllysine-residue acetyltransferase
	*VpaChn25_RS04475*	0.439	ADP-forming succinate–CoA ligase subunit beta
	*VpaChn25_RS04465*	0.485	2-oxoglutarate dehydrogenase E1 component
	*VpaChn25_RS04455*	0.440	Succinate dehydrogenase flavoprotein subunit
	*VpaChn25_RS04440*	0.472	Citrate synthase
Pentose and glucuronate interconversions	*VpaChn25_RS23490*	0.393	L-arabinose isomerase
	*VpaChn25_RS07015*	5.792	Aldehyde dehydrogenase (NADP(+))
	*VpaChn25_RS23600*	0.321	Mannonate dehydratase
	*VpaChn25_RS23625*	0.331	Fructuronate reductase
	*VpaChn25_RS23630*	0.370	Glucuronate isomerase
	*VpaChn25_RS23480*	0.097	Ribulokinase
Butanoate metabolism	*VpaChn25_RS07255*	0.460	4-aminobutyrate-2-oxoglutarate transaminase
	*VpaChn25_RS18405*	2.693	Acetoacetate-CoA ligase
	*VpaChn25_RS16990*	0.104	Aspartate aminotransferase family protein
	*VpaChn25_RS18185*	2.591	Class I poly(R)-hydroxyalkanoic acid synthase
	*VpaChn25_RS01855*	0.394	Acetolactate synthase small subunit
	*VpaChn25_RS01850*	0.436	Acetolactate synthase 3 large subunit
	*VpaChn25_RS10475*	0.470	Bifunctional acetaldehyde-CoA/alcohol dehydrogenase
	*VpaChn25_RS22970*	0.479	Formate C-acetyltransferase/glycerol dehydratase family glycyl radical enzyme
ABC transporters	*VpaChn25_RS20680*	2.168	ABC transporter ATP-binding protein
	*VpaChn25_RS18435*	2.674	Branched-chain amino acid ABC transporter permease
	*VpaChn25_RS15795*	0.489	Amino acid ABC transporter substrate-binding protein
	*VpaChn25_RS07075*	3.318	ATP-binding cassette domain-containing protein
	*VpaChn25_RS07770*	0.320	Tungsten ABC transporter substrate-binding protein
	*VpaChn25_RS23465*	0.252	L-arabinose ABC transporter permease AraH
	*VpaChn25_RS18440*	2.085	Branched-chain amino acid ABC transporter permease
	*VpaChn25_RS18445*	2.037	Branched-chain amino acid ABC transporter substrate-binding protein
	*VpaChn25_RS23380*	0.422	Fe(3+) dicitrate ABC transporter ATP-binding protein FecE
	*VpaChn25_RS22050*	2.141	iron ABC transporter permease
	*VpaChn25_RS23470*	0.108	L-arabinose ABC transporter ATP-binding protein AraG
	*VpaChn25_RS23475*	0.141	Arabinose ABC transporter substrate-binding protein
	*VpaChn25_RS16420*	0.217	ABC transporter ATP-binding protein
	*VpaChn25_RS18450*	2.058	ABC transporter ATP-binding protein
	*VpaChn25_RS16425*	0.257	ABC transporter permease
	*VpaChn25_RS21150*	2.079	sn-glycerol-3-phosphate ABC transporter ATP-binding protein UgpC
	*VpaChn25_RS07085*	5.165	ABC transporter permease subunit
	*VpaChn25_RS07080*	4.177	ATP-binding cassette domain-containing protein
	*VpaChn25_RS20855*	2.741	Transporter substrate-binding domain-containing protein
	*VpaChn25_RS20850*	3.288	Arginine ABC transporter permease ArtQ
	*VpaChn25_RS18745*	0.192	Ribose ABC transporter substrate-binding protein RbsB
	*VpaChn25_RS21135*	0.294	Sugar ABC transporter permease
	*VpaChn25_RS16430*	0.315	ABC transporter ATP-binding protein
	*VpaChn25_RS21145*	0.209	Extracellular solute-binding protein
	*VpaChn25_RS21140*	0.325	Sugar ABC transporter permease
	*VpaChn25_RS07095*	4.736	Peptide ABC transporter substrate-binding protein
	*VpaChn25_RS07090*	5.473	Oligopeptide ABC transporter permease OppB
	*VpaChn25_RS20845*	4.173	Arginine ABC transporter permease ArtM
	*VpaChn25_RS03505*	0.442	MetQ/NlpA family lipoprotein
	*VpaChn25_RS20860*	2.059	Arginine ABC transporter ATP-binding protein ArtP
	*VpaChn25_RS18620*	0.063	Choline ABC transporter substrate-binding protein
	*VpaChn25_RS18625*	0.076	Choline ABC transporter permease subunit
	*VpaChn25_RS18740*	0.362	Ribose ABC transporter permease
	*VpaChn25_RS22165*	0.221	Maltose/maltodextrin ABC transporter substrate-binding protein MalE
	*VpaChn25_RS18630*	0.132	Choline ABC transporter ATP-binding protein
	*VpaChn25_RS17000*	0.498	Putative 2-aminoethylphosphonate ABC transporter substrate-binding protein
	*VpaChn25_RS18730*	0.127	D-ribose pyranase
	*VpaChn25_RS18735*	0.327	Ribose ABC transporter ATP-binding protein RbsA
	*VpaChn25_RS18425*	2.079	ABC transporter ATP-binding protein
Quorum sensing	*VpaChn25_RS14495*	0.459	Protein-export chaperone SecB
	*VpaChn25_RS12590*	2.502	ABC transporter ATP-binding protein
	*VpaChn25_RS15815*	0.388	ABC transporter ATP-binding protein
	*VpaChn25_RS18430*	3.205	Long-chain fatty acid–CoA ligase
	*VpaChn25_RS12580*	2.162	ABC transporter permease
	*VpaChn25_RS12585*	2.557	ABC transporter ATP-binding protein
	*VpaChn25_RS19030*	0.412	Quorum-sensing autoinducer synthase
	*VpaChn25_RS07050*	9.639	ABC transporter permease
	*VpaChn25_RS25650*	0.474	GTP cyc Meng ydrolase II
	*VpaChn25_RS09025*	0.424	Response regulator
	*VpaChn25_RS09695*	0.442	Anthranilate synthase component 1
	*VpaChn25_RS00230*	0.497	ABC transporter permease
	*VpaChn25_RS09030*	0.188	Two-component sensor histidine kinase
	*VpaChn25_RS07030*	6.574	Extracellular solute-binding protein
	*VpaChn25_RS07045*	6.497	ABC transporter ATP-binding protein
	*VpaChn25_RS17545*	0.461	Extracellular solute-binding protein
	*VpaChn25_RS07055*	8.372	ABC transporter permease
	*VpaChn25_RS09700*	0.386	Aminodeoxychorismate/anthranilate synthase component II
Glycerolipid metabolism	*VpaChn25_RS16340*	0.162	Glycerate kinase
	*VpaChn25_RS01910*	0.167	Dihydroxyacetone kinase ADP-binding subunit DhaL
	*VpaChn25_RS11685*	0.034	Glycerol kinase
	*VpaChn25_RS01905*	0.153	Dihydroxyacetone kinase subunit DhaK
	*VpaChn25_RS01900*	0.041	Glycerol dehydrogenase
Phosphotransferase system	*VpaChn25_RS16970*	0.353	PTS sugar transporter subunit IIB
	*VpaChn25_RS10110*	0.199	PTS glucose transporter subunit IIBC
	*VpaChn25_RS03530*	0.416	PTS trehalose transporter subunit IIBC
	*VpaChn25_RS04190*	0.487	Phosphoenolpyruvate-protein phosphotransferase PtsI
	*VpaChn25_RS13660*	0.473	HPr family phosphocarrier protein
Valine, leucine and isoleucine degradation	*VpaChn25_RS18590*	2.510	3-hydroxyisobutyrate dehydrogenase
	*VpaChn25_RS18535*	2.274	Hydroxymethylglutaryl-CoA lyase
	*VpaChn25_RS18570*	2.783	CoA-acylating methylmalonate-semialdehyde dehydrogenase
	*VpaChn25_RS18550*	2.559	Methylcrotonoyl-CoA carboxylase
	*VpaChn25_RS18540*	2.521	Acetyl/propionyl/methylcrotonyl-CoA carboxylase subunit alpha
Thiamine metabolism	*VpaChn25_RS15465*	0.279	Phosphomethylpyrimidine synthase ThiC
	*VpaChn25_RS15460*	0.171	Thiamine phosphate synthase
	*VpaChn25_RS16435*	0.420	Bifunctional hydroxymethylpyrimidine kinase/phosphomethylpyrimidine kinase
	*VpaChn25_RS16405*	0.310	Thiamine phosphate synthase
	*VpaChn25_RS15455*	0.158	Thiazole biosynthesis adenylyltransferase ThiF
	*VpaChn25_RS16410*	0.289	Hydroxyethylthiazole kinase
	*VpaChn25_RS16415*	0.244	Thiaminase II
	*VpaChn25_RS15445*	0.134	Thiazole synthase
	*VpaChn25_RS15440*	0.163	2-iminoacetate synthase ThiH

Remarkably, approximately sixty DEGs involved in the galactose metabolism, fructose and mannose metabolism, TCA, glycerolipid metabolism, PTS, and thiamine metabolism were all significantly down-regulated in the *ΔVpaChn25_0724* mutant (0.034- to 0.487-fold) (p<0.05), when compared with the wild type and *ΔVpaChn25_0724-com* strains (**Table 3**). Also, all the DEGs in the pentose and glucuronate interconversions were greatly down-regulated (0.097- to 0.393-fold), except one encoding aldehyde dehydrogenase. These changes were directly related to the observed phenotypic variations of *ΔVpaChn25_0724*. For example, in the galactose metabolism, eleven DEGs encoding key metabolizing enzymes were significantly repressed at the transcriptional level (0.171–0.441 fold) (p<0.05), including the galactokinase (*VpaChn25*_*RS11750* and *VpaChn25*_*RS21100*), galactose-1-epimerase (*VpaChn25_RS11745*), alpha and beta-galactosidase (*VpaChn25*_*RS21125*, *VpaChn25*_*RS06235*, *VpaChn25*_*RS11770* and *VpaChn25*_*RS11775*), UDP-glucose 4-epimerase GalE (*VpaChn25_RS21110* and *VpaChn25_RS11760*), and UDP-glucose-hexose-1-phosphate uridylyltransferase (*VpaChn25*_*RS11755* and *VpaChn25*_*RS21105*), which may have resulted in reduced important metabolites (e.g., galactose-1-phosphate, and UDP-glucose/galactose/fructose 1,6-diphosphate) in the metabolic pathway. Similarly, in the fructose and mannose metabolism, the DEGs encoding key metabolizing enzymes, e.g., 1-phosphofructokinase (*VpaChn25*_*RS19525*) and mannose-6-phosphate isomerase (*VpaChn25*_*RS22285*, *VpaChn25*_*RS20215*), were also significantly down-regulated (0.156- to 0.326-fold) (p<0.05). Moreover, approximately all the DEGs linked to PTS showed a significant decrease in transcription in *ΔVpaChn25_0724* (0.191- to 0.473-fold) (p<0.05). Interestingly, expression of the gene encoding glucose transporter subunit IIBC (*VpaChn25*_*RS10110*) was notably down-regulated (0.199-fold). Meanwhile, expression of about nine DEGs involved in TCA was slightly down-regulated (0.365- to 0.486-fold) (p<0.05). In addition, in the thiamine metabolism, expression of thiamine phosphorylase (*VpaChn25_RS16405*) was also down-regulated (0.310-fold) (p<0.05). These data suggested inactive transport and utilization of the carbon sources as well as repressed energy production in the *ΔVpaChn25_0724* mutant.

Additionally, five DEGs involved in the glycerolipid metabolism were strikingly down-regulated (0.034- to 0.041-fold) (p<0.05), including the glycerol kinase (*VpaChn25*_*RS16340* and *VpaChn25*_*RS11685*), glycerol dehydrogenase (*VpaChn25*_*RS01900*), dihydroxyacetone kinase ADP-binding subunit DhaL (*VpaChn25*_*RS01910*), and dihydroxyacetone kinase subunit DhaK genes (*VpaChn25*_*RS01905*). The reaction product (glycerol 3-phosphate, G3P) catalyzed by glycerol kinase is also an important metabolite in phospholipid biosynthesis under all growth conditions (Holtman et al., 2001), which plays a vital role in the regulation of membrane biogenesis.

On the other aspect, the deletion of *VpaChn25_0724* also triggered significant changes in the other five metabolic pathways in the *ΔVpaChn25_0724* mutant. Many DEGs thereof showed higher transcriptional levels. For instance, most DEGs linked to the valine, leucine and isoleucine degradation were slightly up-regulated in *ΔVpaChn25_0724* (2.179- to 2.783-fold) (p<0.05), which may have resulted in an increases in acetyl-CoA and subsequent entry into TCA. Remarkably, approximately 39 DEGs associated with ABC transporters were significantly altered at the transcriptional level. Consistent with the down-regulated carbon metabolism as well as repressed energy production in *ΔVpaChn25_0724*, some ABC transporters for sugar uptake were also greatly down-regulated (0.192- to 0.362-fold) (p<0.05), e.g., sugar ABC transporter permease (*VpaChn25*_*RS21140* and *VpaChn25*_*RS21135*), maltose/maltodextrin ABC transporter substrate-binding protein MalE (*VpaChn25*_RS22165), ribose ABC transporter permease (*VpaChn25*_*RS18740*), ribose ABC transporter ATP-binding protein RbsAB (*VpaChn25*_*RS18735* and *VpaChn25*_*RS187450*), and extracellular solute-binding protein (*VpaChn25*_*RS21145*). In marked contrast, the DEGs encoding arginine ABC transporter permease ArtM (*VpaChn25*_*RS20845*), peptide ABC transporter substrate-binding protein (*VpaChn25*_*RS07095*), and oligopeptide ABC transporter permease (*VpaChn25*_*RS07090*) were notably up-regulated (4.173- to 5.473-fold). Likewise, some DEGs encoding ABC transporter ATP-binding protein (*VpaChn25*_*RS07045*), ABC transporter permease (*VpaChn25*_*RS07085*, *VpaChn25*_*RS07050* and *VpaChn25*_*RS07055*), and extracellular solute-binding protein (*VpaChn25*_*RS07030*) were also greatly up-regulated in the QS (5.165- to 9.639-fold) (p<0.05), suggesting that the *VpaChn25_0724* gene may act as a suppressor of these ABC transporters in *V. parahaemolyticus* CHN25.

### Major Altered DGEs Related with Phenotypic Variations of the ΔVpaChn25_0724 Mutant

On the basis of the transcriptome data, the GSAA against the GO database also revealed major altered DGEs related with phenotypic variations of the Δ*VpaChn25_0724* mutant compared with the wild type and Δ*VpaChn25_0724-com* strain.

Expression of the DEGs encoding flagellar basal body protein FliL (*VpaChn25_RS22910*) (0395-fold), rod protein FlgD (*VpaChn25_RS17140*) (0.389-fold), and export and assembly protein FliR (*VpaChn25_RS22850*) (0.094-fold) were all significantly down-regulated in the Δ*VpaChn25_0724* mutant (p<0.05). The remarkably repressed FliR (0.094-fold) belongs to a membrane-embedded part of flagellar export apparatus. These data suggested a defective flagellar basal body in the Δ*VpaChn25_0724* mutant that may have contributed to its affected swimming and biofilm formation.

Some biofilm formation-associated genes were also repressed at the transcriptional level in the Δ*VpaChn25_0724* mutant, e.g., QS autoinducer synthase (*VpaChn25_RS19030*), anthranilate synthase component 1 (*VpaChn25_RS09695*), aminodeoxychorismate/anthranilate synthase component II (*VpaChn25_RS09700*), and PTS glucose transporter subunit IIBC (*VpaChn25_RS10110*) (0.199- to 0.442-fold) (p<0.05). QS impacts bacterial motility, biofilm formation, and construction (Whiteley et al., 2017). The conserved phage shock protein (Psp) system functions in cell envelope stress response, and links to antibiotic resistance, biofilm formation and virulence in a diverse group of bacteria (Flores-Kim and Darwin, 2016). In this study, the genes encoding core components of the Psp system PspB (*VpaChn25_RS06290*) (0.385-fold) and PspC (*VpaChn25_RS06295*) (0.342-fold) were significantly down-regulated in the Δ*VpaChn25_0724* mutant (p<0.05).

The genes encoding T3SS chaperones SycN (*VpaChn25_RS08695*) (0.197-fold) and YopN-like gatekeeper (*VpaChn25_RS08705*) (0.399-fold), YopR-like regulator (*VpaChn25_RS08820*) (0.461-fold), and export apparatus protein (*VpaChn25_RS08740*) (0.244-fold) were all greatly down-regulated in Δ*VpaChn25_0724* (p<0.05). Additionally, expression of the genes encoding OmpA (*VpaChn25_RS22825*) (0.164-fold) and OmpW (*VpaChn25_RS16240)* (0.331-fold) were significantly decreased (p<0.05). In contrast, the gene encoding T3SS chaperone CesT (*VpaChn25_RS08785*) was notably up-regulated in the Δ*VpaChn25_0724* mutant (6.221-fold) (p<0.05).

**Figure 8:**
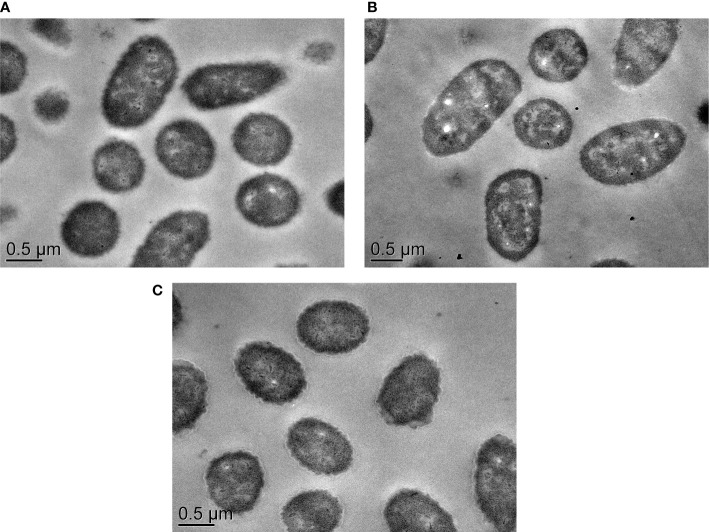
The TEM observation of cell structure of *V. parahaemolyticus* CHN25, Δ*VpaChn25_0724*, and Δ*VpaChn25_0724-com* strains. **(A)** Wild type; **(B)** Δ*VpaChn25_0724*; **(C)** Δ*VpaChn25_0724*-com.

Remarkably, several differentially expressed response and transcriptional regulators were greatly repressed in the Δ*VpaChn25_0724* mutant, which are key components in bacterial gene regulatory networks, and can sense fluctuations under internal and external conditions (Brinkrolf et al., 2007). For example, two genes (*VpaChn25*_*RS08860* and *VpaChn25*_*RS22275*) encoded DNA-binding transcriptional regulator AraC, one of the most common positive regulators in bacteria, were notably down-regulated (0.270-fold and 0.204-fold) (p<0.05). Regulators belonging to this family have three major regulatory functions in common: carbon metabolism, stress response, and pathogenesis (Gallegos et al., 1997). Moreover, the gene (*VpaChn25*_*RS04915*) encoding a TetR/AcrR family transcriptional regulator was strikingly down-regulated (0.021-fold) (p<0.05). Another interesting observation was that expression of a regulator BetI (*VpaChn25*_*RS18605*) was also strikingly down-regulated in the Δ*VpaChn25_0724* mutant (0.022-fold) (p<0.05). Additionally, TEM images provided additional evidence for the global-level gene expression change in *ΔVpaChn25_0724*, as cell morphological characteristics of this mutant appeared different from those of the wild type and Δ*VpaChn25_0724*-com strains, such as the changed intracellular structure with more cytocysts in *ΔVpaChn25_0724* (**Figure 8**).

On the other hand, to further verify the differential transcriptomic data, two representative DEGs *Vpachn25_RS01720* and *Vpachn25_RS04440* (**Table S1**) encoding key enzymes were chosen for enzyme activity analyses, given their detection methods are available in literature to date. The results showed significantly reduced malate dehydrogenase and citrate synthase activities in *ΔVpaChn25_0724* compared with the wild type strain (p<0.05) (**Figure S2**), which were encoded by the *Vpachn25_RS01720* and *Vpachn25_RS04440*, respectively. These results were correlated with those yielded from the transcriptome analysis and confirmed by the RT-qPCR analyses (**Table S1**).

On the basis of the findings, we tried to locate the protein encoded by *VpaChn25_0724* in *V. parahaemolyticus* CHN25 using routine immunochemistry method, but failed to find its exact cellular position. It will be interesting to investigate its protein property and regulation in the bacterium in the future research.

### Transmission of the VpaChn25_0724 Gene in Bacteria

The *VpaChn25_0724* gene was examined in 138 V*. parahaemolyticus* strains isolated from aquatic products collected in Shanghai, China (Su et al. under review) by PCR assay. The resulting data showed that approximately 5.8% (n=8) of the *V. parahaemolyticus* isolates carried the *VpaChn25_0724* gene. Moreover, BLAST analysis against the GenBank database revealed that the *VpaChn25_0724* gene is present in three *Vibrio* phages, four *Vibrio* species including *Vibrio cholera*e, *Vibrio campbellii, Vibrio mimicus*, and *V. parahaemolyticus*, as well as the other two bacterial genera such as *Marinomonas primoryensis*, and *Shewanella oneidensis*. These data indicated that transmission of the *VpaChn25_0724* gene occurred among *V. parahaemolyticus* population in aquatic products, within *Vibrio* genus, and even across bacterial genera during the evolution history.

## Discussion

*Vibrio parahaemolyticus* is a leading seafood-borne pathogen worldwide. In aquatic ecosystems, phages constantly transform bacterial communities by HGT (Penades et al., 2015; Castillo et al., 2018). Nevertheless, biological functions of prophage-related genes remaining in *V. parahaemolyticus* are not yet fully understood. In this study, for the first time, we studied one such gene *VpaChn25_0724* encoding an unknown hypothetical protein in the largest prophage gene cluster identified in *V. parahaemolyticus* CHN25 genome (Zhu et al., 2017). An unmarked in-frame gene deletion mutant Δ*VpaChn25_0724* was successfully constructed, and its complementary mutant Δ*VpaChn25_0724*-com was also obtained in this study. Our data unveiled that the *VpaChn25_0724* gene deletion resulted in a sever defect in growth of *V. parahaemolyticus* CHN25, particularly at the lower temperatures.

Motility is closely associated with bacterial virulence and affects their attachment, colonization and invasion toward host cells (Guo et al., 2019). *V. parahaemolyticus* is motile by means of a single, sheathed polar flagellum that propels the swimmer cell in liquid environments (Kim and McCarter, 2000). The bacterial flagellum is essential in forming biofilm (i.e., matrix enclosed and surface-associated communities), which is critical for *V. parahaemolyticus* persistence in aquatic environments and pathogenicity in the host (Yildiz and Visick, 2009). In this study, we observed significantly lowered swimming capacity of the Δ*VpaChn25_0724* mutant at 25°C and 15°C, when compared with the wild type (*p* < 0.05). Moreover, our data showed a decrease in the development of biofilm by *V. parahaemolyticus* CHN25 in the absence of the *VpaChn25_0724* gene. The severe defect in growth elicited by the *VpaChn25_0724* gene deletion likely led to the variant swimming motility and biofilm formation of *V. parahaemolyticus* CHN25.

Bacterial secretion systems play a vital role in virulence, symbiosis, interbacterial interactions, and environmental stress (De Nisco et al., 2017). In this study, we found differential secretomes mediated by the *VpaChn25_0724* gene deletion. Among the differentially expressed extracellular proteins, the protein Spot 24-b-1 is an important outer membrane protein and protects bacteria against host phagocytosis (Li et al., 2016). The protein Spots 24-b-2 and 24-b-3, identified as FlaB/D and FlaA flagellins, respectiely, are involved in polar flagellar biosynthesis in *V. parahaemolyticus* (Kim and McCarter, 2000). The latter is involved in P-ring assembly of flagellar structure and swimming motility (Kim and McCarter, 2000). Recently, Echazarreta et al. reported that FlaA also facilitated filament formation of *V. cholerae* flagellum (Echazarreta et al., 2018). The protein Spot 24-b-6 was identified as an AdhE that forms a high-order spirosome architecture for its activity (Kim et al., 2019). This multi-functional enzyme is essential for the fermentation of glucose to sustain the glycolytic pathway, and the deletion of *adhE* gene in pathogenic *E. coli* O157:H7 strongly suppressed type III secretion systems (T3SS) and induced over-expression of non-functional flagella (Kim et al., 2019). The Spot 24-C-1, identified as a 2-hydroxyacid dehydrogenase, plays an important role in cell stability at high saline concentrations (Bonete et al., 2000). These results indicated that the *VpaChn25_0724* gene deletion resulted in increased numbers of extracellular proteins, which was likely associated with damaged cell membrane of the Δ*VpaChn25_0724* mutant.

The damaged cell membrane elicited by the *VpaChn25_0724* gene deletion also significantly affected *V. parahaemolyticus* CHN25-host intestinal epithelial cell interaction. Based on the *in vitro* Caco-2 cell model, our data showed that the *ΔVpaChn25_0724* mutant induced much higher viability and early apoptosis rate of Caco-2 cells than the wild type and the Δ*VpaChn25_0724*-com strains (*p* < 0.01). Nevertheless, an opposite pattern was observed in the late apoptosis elicited by *ΔVpaChn25_0724*. The cell membrane of Caco-2 cells at the early apoptosis phase was still intact, whereas at the later stage was damaged. The most possible explanation for the above observation was that the increased number of extracellular proteins secreted by the *ΔVpaChn25_0724* mutant may have contributed to the higher apoptosis occurrence at the early stage, however, the significantly changed membrane surface structure and cellular process (see below) of the mutant likely lowered its cytotoxicity to the host cells at the later stage.

Transcriptomes of *V. parahaemolyticus* CHN25, *ΔVpaChn25_0724* and Δ*VpaChn25_0724*-com strains were determined to get insights into global-level gene expression change mediated by the *VpaChn25_0724* gene deletion. Based on the GSEA of the transcriptome data against the KEGG database, approximately twelve significantly altered metabolic pathways were identified in the *VpaChn25_0724* mutant (**Table 3**). For instance, PTS is known as a major sugar transport multicomponent system in bacteria, by which many sugars are transported into bacteria, concomitantly phosphorylated, and then fed into glycolysis (Postma et al., 1993). In this study, approximately all the DEGs linked to PTS showed a significant decrease in transcription in *ΔVpaChn25_0724* (0.191- to 0.473-fold) (p<0.05), which encode fructose, glucose. mannitol, trehalose, and sugar transporter subunits. Moreover, expression of the gene encoding glucose transporter subunit IIBC (*VpaChn25*_*RS10110*) was notably down-regulated (0.199-fold), which may be related with the repressed galactose, fructose, mannose and glycerol metabolisms, because glucose controls utilization of several other carbon sources including lactose, melibiose, maltose, and glycerol in *E. coli* (Holtman et al., 2001). Meanwhile, expression of about nine DEGs involved in TCA was slightly down-regulated (p<0.05). In addition, in the thiamine metabolism, expression of thiamine phosphorylase (*VpaChn25_RS16405*) was also down-regulated (p<0.05), which may result in decreased thiamine pyrophosphate, a cofactor for many essential enzymes in glucose and energy metabolisms (Rodionov et al., 2017). These data suggested inactive transport and utilization of the carbon sources as well as repressed energy production in the *ΔVpaChn25_0724* mutant.

On the basis of the transcriptome data, major altered DGEs related with the phenotypic variations of the Δ*VpaChn25_0724* mutant were also identified. For instance, bacterial polar flagellum is powered by a rotary motor and acts as semirigid helical propeller, which is attached *via* a flexible coupling, known as the hook, to the basal body. The latter consists of rings and rods that penetrate the membrane and peptidoglycan layers (Kim and McCarter, 2000). In this study, expression of the DEGs encoding flagellar basal body structure protein FliL (*VpaChn25_RS22910*), rod structure protein FlgD (*VpaChn25_RS17140*), and export and assembly structure protein FliR (*VpaChn25_RS22850*) were all significantly down-regulated in the Δ*VpaChn25_0724* mutant (p<0.05). Recently, Takekawa et al. reported that FliL is a new stomatin-like protein that assists the *Vibrio* flagellar motor function (Takekawa et al., 2019). In this study, the FliR that belongs to a membrane-embedded part of flagellar export apparatus was greatly down-regulated in expression (0.094-fold). These data suggested a defective flagellar basal body in the Δ*VpaChn25_0724* mutant that may have contributed to its affected swimming and biofilm formation.

T3SS was identified in *V. parahaemolyticus* CHN25 genome, which is necessary for bacterial survival in the environment (De Nisco et al., 2017; Matsuda et al., 2019). In this study, the genes encoding T3SS chaperones SycN (*VpaChn25_RS08695*) and YopN-like gatekeeper (*VpaChn25_RS08705*), YopR-like regulator (*VpaChn25_RS08820*), and export apparatus protein (*VpaChn25_RS08740*) were all greatly down-regulated in Δ*VpaChn25_0724* (p<0.05). In *Yersinia pestis*, the secretion of toxic *Yersinia* outer proteins (Yops) is regulated by a YopN/SycN/YscB/TyeA complex (Joseph and Plano, 2013). It has been reported that YopN functions to prevent the secretion of Yops until T3SS apparatuses are in direct contact with a targeted eukaryotic cell and activated, which avoids the spurious loss of effector proteins to the extracellular environment (Plano and Schesser, 2013). Additionally, expression of the genes encoding OmpA (*VpaChn25_RS22825*) and OmpW (*VpaChn25_RS16240)* were significantly decreased (p<0.05). OmpA family proteins are heat-modifiable, surface-exposed, and porin proteins that are in high-copy number in the outer membrane of many gram-negative pathogenic bacteria. They are involved in bacterial adhesion, invasion or intracellular survival, as well as evasion of host defenses or stimulation of pro-inflammatory cytokine production (Confer and Ayalew, 2013). In this study, in contrast, the gene encoding T3SS chaperone CesT (*VpaChn25_RS08785*) was notably up-regulated in the Δ*VpaChn25_0724* mutant (6.221-fold) (p<0.05). It has been reported that the enteropathogenic *E. coli* (EPEC) multicargo chaperone CesT interacts with at least ten effector proteins and contributes pathogenesis (Ramu et al., 2013).

The distinct transcriptome data also revealed strikingly down-regulated five key genes involved in the glycerolipid metabolism in the *ΔVpaChn25_0724* mutant (0.034- to 0.041-fold), suggesting reduced important metabolites in the bacterial phospholipid biosynthesis, such as the G3P (Holtman et al., 2001) that plays a vital role in the regulation of membrane biogenesis. Meanwhile, a defective flagellar basal body in Δ*VpaChn25_0724* was also revealed by the comparative transcriptomic analyses (see above). These results, coupled with the inactive transport and utilization of the carbon sources as well as repressed energy production in *ΔVpaChn25_0724* may have significantly affected the cell membrane integrity of the mutant.

In addition, several key components in bacterial gene regulatory networks were greatly repressed in the Δ*VpaChn25_0724* mutant, which can sense fluctuations under internal and external conditions (Brinkrolf et al., 2007). For instance, the gene (*VpaChn25*_*RS04915*) encoding a TetR/AcrR family transcriptional regulator was strikingly down-regulated (0.021-fold) (p<0.05). Regulators of this family are involved in a series of regulatory cascades, e.g., cell response to environmental insults, control of catabolic pathways, differentiation processes, and pathogenicity (Ramos et al., 2005). Another interesting observation was that expression of a regulator BetI (*VpaChn25*_*RS18605*) was also strikingly down-regulated in the Δ*VpaChn25_0724* mutant (0.022-fold) (p<0.05), which negatively regulates *betT* and *betIBA* genes that govern glycine betaine (GB) biosynthesis from choline in *E. coli* (Rkenes et al., 1996). The down-regulated BetI possibly in turn activated the target genes in Δ*VpaChn25_0724*, which perhaps led to increased amount of GB to maintain the integrity of cell membranes against the damaging effects, as in other stress responses to excessive salt, cold, heat and freezing in bacteria (Sun et al., 2014).

Taken together, the results in this study facilitate better understanding of biological function of prophage-related genes remaining in *V. parahaemolyticus*, and meet the increasing need for novel diagnosis candidates of the leading seafood-borne pathogen worldwide.

## Data Availability Statement

A complete list of the DEGs is available in the NCBI SRA database (http://www.ncbi.nlm.nih.gov/sra/) under the accession number SRP258529.

## Author Contributions

LY, YW, PY, SR, ZZ, YJ, JY, XP, and LC participated in the design and or discussion of the study. LY, YW, and ZZ carried out the major experiments. PY analyzed the data. SR supervised this study. JY helped the bacteria-host intestinal epithelial cell interaction experiments. LY, YW, ZZ, and LC wrote the manuscript. XP, and LC revised the manuscript. All authors contributed to the article and approved the submitted version.

## Funding

This study was supported by grants from the Science and Technology Commission of Shanghai Municipality (No. 17050502200) and the National Natural Science Foundation of China (No. 31671946).

## Conflict of Interest

The authors declare that the research was conducted in the absence of any commercial or financial relationships that could be construed as a potential conflict of interest.

## Supplementary Material

Supplementary Figure 1 Construction of the Δ*VpaChn25_0724*, and Δ*VpaChn25_0724-com* mutants by agarose gel electrophoresis analysis. **(A)** amplicons of upstream (Lane 1) and downstream (Lane 2) sequences of the *VpaChn25_0724* gene. **(B)** amplicons franking the upstream and downstream sequence of *VpaChn25_0724*. **(C)** amplicons of the upstream and downstream sequence of *VpaChn25_0724* in the recombinant pDS132+*VpaChn25_0724* plasmids. Lines 1–6, positive recombinant plasmids; Line 7, blank control; Line 8, positive control. **(D)** amplicons of transformants with the *VpaChn25_0724*-up-F and *VpaChn25_0724*-down-R primers. Lines 1–5, positive strain of the first change; Lines 6–7, positive control; Lines 8 to 9, blank control. **(E)** amplicons of the *tlh* gene. Lines 1–5, positive exconjugants; Line 6, positive control; Line 7, blank control. **(F)** amplicons of exconjugants with the *VpaChn25_0724*-up-ex-F and *VpaChn25_0724*-down-ex-R primers. Lines 1–22, selected exconjugants strains; Line 23, blank control; Line 24, genomic DAN control. **(G)** Amplicons of the *tlh* gene from Δ*VpaChn25_0724* mutants. Lines 1–5, positive Δ*VpaChn25_0724* mutants; Line 6, positive control; Line 7, blank control. **(H)** Amplicons of the *VpaChn25_0724* gene. Lines 1–2, positive bands; Line 3, blank control. **(I)** double digestion of the recombinant pMMB207+*VpaChn25_0724* plasmid. **(J)** amplicons of *VpaChn25_0724* from positive transformants. **(K)** amplicons of *tlh from* Δ*VpaChn25_0724-com* mutants. M, 100 bp DNA Ladder.

Supplementary Figure 2 Activities of the malate dehydrogenase and citrate synthase encoded by the DEGs *Vpachn25_RS01720* and *Vpachn25_RS04440* in *V. parahaemolyticus* CHN25 (WT), Δ*VpaChn25_0724*, and Δ*VpaChn25_0724*-com strains. A. Malate dehydrogenase. B. Citrate synthase.

Supplementary Table S1 Expression of representative DEGs in ΔVpaChn25_0724 mutant by RT-qPCR analysis.
