# Supplementary figures and images for: Prophage-Related Gene VpaChn25_0724 Contributes to Cell Membrane Integrity and Growth of Vibrio parahaemolyticus CHN25
Source: Front Cell Infect Microbiol. 2020 Dec 9;10:595709. doi: 10.3389/fcimb.2020.595709 (PMC7756092; doi:10.3389/fcimb.2020.595709)

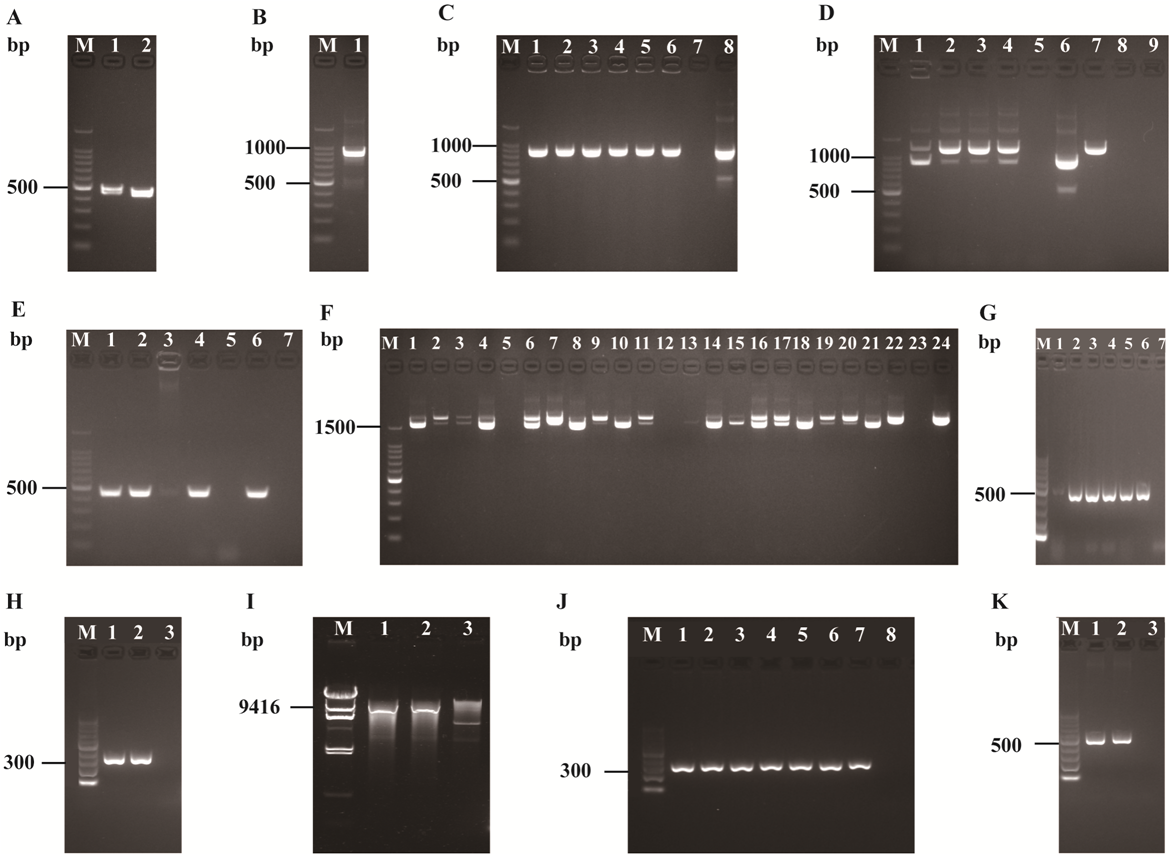

Supplement: Supplementary Figure 1 — Construction of the ΔVpaChn25_0724, and ΔVpaChn25_0724-com mutants by agarose gel electrophoresis analysis. (A) amplicons of upstream (Lane 1) and downstream (Lane 2) sequences of the VpaChn25_0724 gene. (B) amplicons franking the upstream and downstream sequence of VpaChn25_0724. (C) amplicons of the upstream and downstream sequence of VpaChn25_0724 in the recombinant pDS132+VpaChn25_0724 plasmids. Lines 1–6, positive recombinant plasmids; Line 7, blank control; Line 8, positive control. (D) amplicons of transformants with the VpaChn25_0724-up-F and VpaChn25_0724-down-R primers. Lines 1–5, positive strain of the first change; Lines 6–7, positive control; Lines 8 to 9, blank control. (E) amplicons of the tlh gene. Lines 1–5, positive exconjugants; Line 6, positive control; Line 7, blank control. (F) amplicons of exconjugants with the VpaChn25_0724-up-ex-F and VpaChn25_0724-down-ex-R primers. Lines 1–22, selected exconjugants strains; Line 23, blank control; Line 24, genomic DAN control. (G) Amplicons of the tlh gene from ΔVpaChn25_0724 mutants. Lines 1–5, positive ΔVpaChn25_0724 mutants; Line 6, positive control; Line 7, blank control. (H) Amplicons of the VpaChn25_0724 gene. Lines 1–2, positive bands; Line 3, blank control. (I) double digestion of the recombinant pMMB207+VpaChn25_0724 plasmid. (J) amplicons of VpaChn25_0724 from positive transformants. (K) amplicons of tlh from ΔVpaChn25_0724-com mutants. M, 100 bp DNA Ladder. [file Image_1.tif]

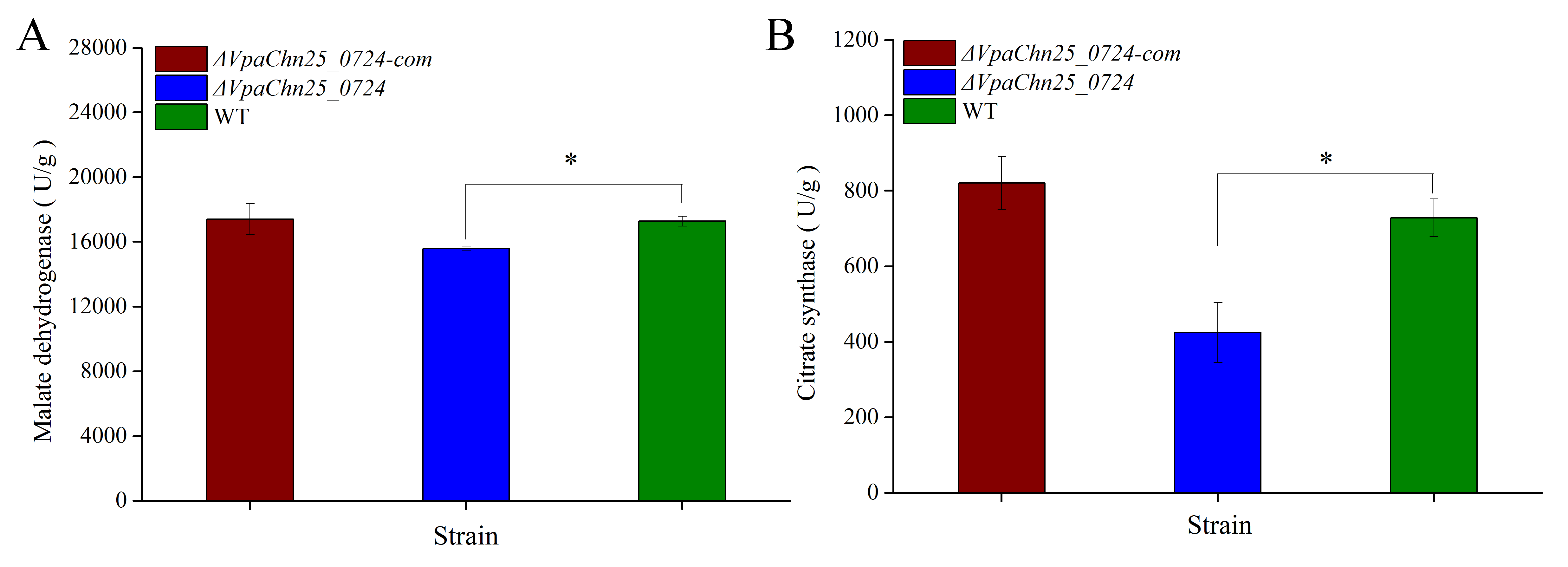

Supplement: Supplementary Figure 2 — Activities of the malate dehydrogenase and citrate synthase encoded by the DEGs Vpachn25_RS01720 and Vpachn25_RS04440 in V. parahaemolyticus CHN25 (WT), ΔVpaChn25_0724, and ΔVpaChn25_0724-com strains. A. Malate dehydrogenase. B. Citrate synthase. [file Image_2.tif]
