# Supplementary material for: Prophage-Related Gene VpaChn25_0724 Contributes to Cell Membrane Integrity and Growth of Vibrio parahaemolyticus CHN25
Source: Front Cell Infect Microbiol. 2020 Dec 9;10:595709. doi: 10.3389/fcimb.2020.595709 (PMC7756092; doi:10.3389/fcimb.2020.595709)
Supplement: Supplementary Table S1 — Expression of representative DEGs in ΔVpaChn25_0724 mutant by RT-qPCR analysis. [file Table_1.doc]

**Table S1.** Expression of representative DEGs in Δ*VpaChn25_0724* mutant by RT-qPCR assay

| Gene | Predicted protein | Fold change | |
| --- | --- | --- | --- |
|  |  | RNA-Seq. | RT-PCR |
| *VvpaChn25_RS01720* | Malate dehydrogenase | 0.383 | 0.004 |
| *Vpachn25_RS01900* | Glycerol dehydrogenase | 0.041 | 0.149 |
| *VvpaChn25_RS03850* | Hypothetical protein | 0.000 | 0.000 |
| *VvpaChn25_RS04440* | Citrate synthase | 0.427 | 0.022 |
| *Vpachn25_RS04915* | TetR/AcrR family transcriptional regulator | 0.021 | 0.027 |
| *VvpaChn25_RS06735* | OmpA family protein | 0.972 | 0.288 |
| *Vpachn25_RS07030* | Extracellular solute-binding protein | 6.574 | 2.137 |
| *Vpachn25_RS07045* | ABC transporter ATP-binding protein | 6.497 | 1.381 |
| *Vpachn25_RS07050* | ABC transporter permease | 9.638 | 1.145 |
| *VvpaChn25_RS07910* | Universal stress protein UspE | 0.414 | 0.030 |
| *VvpaChn25_RS08070* | Cell division protein ZapC | 0.602 | 0.049 |
| *Vpachn25_RS08785* | Type III secretion chaperone CesT | 6.221 | 1.329 |
| *VvpaChn25_RS08820* | YopR family type III secretion effector | 0.459 | 0.270 |
| *VvpaChn25_RS11070* | Flagellin | 0.407 | 0.203 |
| *Vpachn25_RS11685* | Glycerol kinase | 0.034 | 0.006 |
| *VvpaChn25_RS13780* | MSHA biogenesis protein MshF | 0.746 | 0.002 |
| *VvpaChn25_RS14070* | DNA-bindingtranscriptional regulator OxyR | 0.474 | 0.297 |
| *Vpachn25_RS18605* | Transcriptional regulator BetI | 0.022 | 0.028 |
